# Identification of host proteins interacting with the E protein of porcine epidemic diarrhea virus

**DOI:** 10.3389/fmicb.2024.1380578

**Published:** 2024-03-21

**Authors:** Yingwu Qiu, Yingshuo Sun, Xiaoyu Zheng, Lang Gong, Liangyu Yang, Bin Xiang

**Affiliations:** ^1^College of Veterinary Medicine, Yunnan Agricultural University, Kunming, China; ^2^College of Veterinary Medicine, South China Agricultural University, Guangzhou, China

**Keywords:** porcine epidemic diarrhea virus, E protein, NAD-IDH-β, RPB9, MRNP 41

## Abstract

**Introduction:**

Porcine epidemic diarrhea (PED) is an acute, highly contagious, and high-mortality enterophilic infectious disease caused by the porcine epidemic diarrhea virus (PEDV). PEDV is globally endemic and causes substantial economic losses in the swine industry. The PEDV E protein is the smallest structural protein with high expression levels that interacts with the M protein and participates in virus assembly. However, how the host proteins interact with E proteins in PEDV replication remains unknown.

**Methods:**

We identified host proteins that interact with the PEDV E protein using a combination of PEDV E protein-labeled antibody co-immunoprecipitation and tandem liquid-chromatography mass-spectroscopy (LC-MS/MS).

**Results:**

Bioinformatical analysis showed that in eukaryotes, ribosome biogenesis, RNA transport, and amino acid biosynthesis represent the three main pathways that are associated with the E protein. The interaction between the E protein and isocitrate dehydrogenase [NAD] β-subunit (NAD-IDH-β), DNA-directed RNA polymerase II subunit RPB9, and mRNA-associated protein MRNP 41 was validated using co-immunoprecipitation and confocal assays. NAD-IDH-β overexpression significantly inhibited viral replication.

**Discussion:**

The antiviral effect of NAD-IDH-β suggesting that the E protein may regulate host metabolism by interacting with NAD-IDH-β, thereby reducing the available energy for viral replication. Elucidating the interaction between the PEDV E protein and host proteins may clarify its role in viral replication. These results provide a theoretical basis for the study of PEDV infection mechanism and antiviral targets.

## 1 Introduction

Coronaviruses infect humans, a wide range of mammals, and birds, posing a substantial threat to public health. Porcine epidemic diarrhea virus (PEDV) is an enveloped, single-stranded, positive-sense RNA virus that belongs to the coronavirus family (Xu et al., 2020). PEDV is one of the main pathogens affecting the health of pigs, causing infections and inducing intestinal diseases in pigs of all ages, presenting symptoms such as vomiting and watery diarrhea (Zhai et al., 2023). PEDV was first reported in the United Kingdom in 1971, with the classic strain first isolated and identified in Belgium in 1978 (Pensaert and de Bouck, 1978). Ever since, PEDV outbreaks occur annually worldwide, leading to serious economic losses in the pig industry (Jung and Saif, 2015; Zhang et al., 2023).

The PEDV genome is approximately 28 kb long, containing a 5′ untranslated region, a 3′ untranslated region, seven open reading frames, and encoding 16 non-structural proteins and 4 structural proteins (E, M, S, and N), respectively (Lin et al., 2022). The E protein is the smallest among the structural proteins and comprises of three parts: the short amino-terminal hydrophilic region, the α-helical structure (containing the transmembrane region with a length of approximately 25 amino acids), and the long carboxy-terminal region (Torres et al., 2007; Bai et al., 2022). The host KPNA2 protein inhibits PEDV replication by autophagy targeting and degrading viral E proteins (Gao et al., 2023). The PEDV E protein is mainly localized in the endoplasmic reticulum and functions as an interferon-β antagonist by inhibiting RIG-I-mediated signaling (Zheng et al., 2021). Furthermore, the E protein modulates the host immune response, thereby facilitating PEDV replication (Thavorasak et al., 2022). The interaction between E and M proteins maintains the shape of the viral particles and promotes their release (El Omari et al., 2019). The E protein is crucial for the replication process of coronaviruses. Recombinant coronaviruses lacking the E protein exhibit a significant decrease in viral titers or are non-infectious (Cao et al., 2021). Pathogen infection and viral proliferation rely on the interaction between viral and host proteins (Crua Asensio et al., 2017). However, the role of E proteins in viral invasion and replication remains unclear, along with their interactions with the host proteins. In this study, Co-IP-MS technology was used to study the interaction spectrum between PEDV E protein and host protein, providing a theoretical basis for the pathogenesis of PEDV.

## 2 Materials and methods

### 2.1 Cells, viruses, and plasmids

Vero cells and IPI-FX cells were maintained in Dulbecco’s Modified Eagle Medium (DMEM) containing 10% serum at 37°C with 5% CO_2_. The PEDV strain used in this study was FS202201 (Sun et al., 2023), used in this study. The infected cells were maintained in DMEM containing 7 μg/mL trypsin.

Whole genome PEDV and Vero cell RNA was extracted using the Fastagen assay kit (Fastagen, Shanghai, China) according to the manufacturer’s instructions and reverse transcribed into cDNA using Geenstar reverse transcriptase (Geenstar, Guangzhou, China). The target gene fragment was then amplified using a polymerase chain reaction (PCR). The primer sequences are listed in Table 1. pCAGGS-E-HA and pCAGGS-NAD-IDH-β/MRNP 41/RPB9-flag plasmids were constructed by recombining the target genes with the cleaved pCAGGS vector using Vazyme recombinase (C112) (Vazyme, China, Shanghai). All plasmids were validated using sequencing.

**Table 1:** Primer sequences used to construct plasmids.

Primers	Sequences (5′–3′)
pCAGGS-E-HA-F	ATGTCTAACGGTTCTATTCCC
pCAGGS-E-HA-R	AGAAAGTGCTTCATTTAGTCTAA
pCAGGS-NAD-IDH-β-Flag-F	ATGGCGGCACTGAGCGGTGTCCG
pCAGGS-NAD-IDH-β-Flag-R	TGCACCCCCATGGGAGCTAG
pCAGGS-MRNP 41-Flag-F	ATGAGCCTGTTTGGAACAACCT
pCAGGS-MRNP 41-Flag-R	TAAAGCCCCGGAATAAGAAGTAG
pCAGGS-RPB9-Flag-F	ATGGAGCCCGACGGGACCTAC
pCAGGS-RPB9-Flag-R	CACCGCTGGACCGAGTGA

### 2.2 Reagents and antibodies

LipofectamineTM 2000 (11668500) was purchased from Thermo Fisher Scientific (Shanghai, China). Mouse anti-HA monoclonal antibody (M20003) was purchased from Thermo Fisher Scientific. GAPDH, flag, goat anti-mouse IgG (H + L) CoraLite594-conjugated secondary antibody, and IgG (H + L) CoraLite488-conjugated goat anti-rabbit secondary antibody were purchased from Proteintech (Proteintech, Guangzhou, China). IRDye 800CW goat anti-mouse or anti-rabbit IgG was purchased from Licor (Beijing, China).

### 2.3 Transfection

Vero cells and IPI-FX cells were cultured in DMEM medium containing 10% serum to 80% for transfection. Lipofectamine 2000 and plasmid were diluted separately with 100 μL Opti-MEM (Thermo Fisher Scientific) and mixed well. Diluted DNA was added to diluted Lipofectamine 2000 reagent, mixed well and incubated for 5 min at room temperature. After the DNA-lipid complex was added to the cells, they were incubated in DMEM medium containing 10% serum at 37°C, 5% CO_2_ for 24 h and then used for analysis.

### 2.4 Co-immunoprecipitation

The pCAGGS-E-HA plasmid was transfected, and proteins were extracted 24 h later using radioimmunoprecipitation assay (RIPA) lysis buffer (P0013B Biotronix) containing a protease phosphatase inhibitor mixture (P1048 Biotronix). The cells were incubated at 4°C for 15 min with shaking, centrifuged at 15000 × g for 10 min, and the supernatant removed. The cell lysate was added to Anti-HA immunomagnetic beads, which had been washed with tris-buffered saline (TBS) and incubated for 12 h at 4°C on a shaker. The samples were then subjected to mass spectrometry (MS) analysis.

The pCAGGS-E-HA plasmid and target host gene expression plasmids (pCAGGS-NAD-IDH-β-Flag, pCAGGS-MRNP 41- Flag, or pCAGGS-RPB9- Flag) were co-transfected. After 24 h the cell lysate and anti-HA immunomagnetic beads were incubated in a shaker overnight at 4°C. Beads were washed four times with cold phosphate-buffered saline with Tween 20 (PBST). Cell lysates were diluted in 1 × sodium dodecyl-sulfate (SDS) upsampling buffer, heated to 100°C for 5 min, and subjected to SDS-polyacrylamide gel electrophoresis (PAGE).

### 2.5 LC-MS/MS analysis

The anti-HA immunomagnetic beads were centrifuged, and the supernatant was discarded. The beads were washed twice with PBS (200 μL), with the supernatant centrifuged and discarded after each wash. The beads were resuspended in 100 μL NH_4_HCO_3_ solution (50 mmol/L). DL-dithiothreitol (DTT) solution was added to a final concentration of 10 mmol/L, and the beads were reduced in a water bath at 56°C for 1 h. Indole-3-acetic acid (IAA) solution was added to a final concentration of 50 mmol/L, and the reaction was left to react in the dark for 40 min. Trypsin was added to the substrate (1:100 by mass), beads were digested for 4 h at 37°C, the reaction was continued by adding trypsin at the ratio of trypsin to substrate (1:100 by mass), and left to react at 37°C for 16 h. The beads were washed twice with 200 μL of PBS, and the supernatant was discarded after each wash. The reaction was continued by adding trypsin at a mass ratio of 1:100. After digestion, the peptides were desalted using a self-filling desalting column, and the solvent was evaporated in a vacuum centrifuge concentrator at 45°C. The peptides were dissolved in a sample dissolution solution (0.1% formic acid and 2% acetonitrile), vortexed with sufficient shaking, centrifuged at 12000 × g for 10 min at 4°C, and the supernatant was transferred to the sample tube for MS analysis.

The samples were reconstituted in 5–10 μL mobile phase (0.1% formic acid). A nanocolumn was packed with a reversed-phase ReproSil-Pur C18-AQ resin (1.9 μm, 100 Å, Dr. Maisch GmbH, Germany). After equilibration, each sample was loaded onto the column using a sampler. A gradient was formed, and the peptides were eluted with increasing concentrations of solvent B (80% acetonitrile and 0.1% formic acid). The samples were detected using a mass spectrometer, the Q Exactive^™^ Hybrid Quadrupole-Orbitrap^™^ mass spectrometer (Thermo Fisher Scientific, United States), with the relevant parameters (Table 2).

**Table 2:** Parameters used for mass spectrometry analysis.

Mass spectrometry	
Spray voltage	2.2 kV
Capillary temperature	270°C
MS resolution	70,000 at 400 *m*/*z*
MS precursor *m*/*z* range	300.0–1800.0
Product ion scan range	start from m/z 100
Activation type	CID
Min. signal required	1500.0
Isolation width	3.00
Normalized coll. energy	40.0
Default charge state	6
Activation *Q*	0.250
Activation time	30.000
Data dependent MS/MS	Up to top 20 most intense peptide ions from the preview scan in the Orbitrap

### 2.6 Bioinformatic analysis

Raw MS files were analyzed and searched against the target protein database based on the sample species using MaxQuant (1.6.2.10). The parameters were set as follows: the protein modifications were carbamidomethylation (C) (fixed), oxidation (M) (variable), Acetyl (Protein N-term) (variable); the enzyme specificity was set to trypsin; the maximum missed cleavages were set to 2; the precursor ion mass tolerance was set to 20 ppm, and MS/MS tolerance was 20 ppm. Only high confident identified peptides were chosen for downstream protein identification analysis. Uniprot annotation was performed using OmicsBean based on the identified protein IDs. Next, OmicsBean software^1^ provided functional classification annotations for proteins. Kyoto Encyclopedia of Genes and Genomes (KEGG) pathway annotations were analyzed using Kobas 3.0.

### 2.7 Immunofluorescence assay

Cells were co-transfected with the pCAGGS-E-HA plasmid and targeted host gene expression plasmids (pCAGGS-NAD-IDH-β-Flag, pCAGGS-MRNP 41-Flag, or pCAGGS-RPB9-Flag) for 24 h, and fixed in 4% paraformaldehyde for 15 min at room temperature (RT). Membranes were permeabilized with 0.3% Triton X-100 for 15 min at RT. Cells were incubated with specific antibodies overnight at 4°C or 37°C for 2 h. Secondary antibodies CoraLite488 goat anti-mouse and CoraLite594 goat anti-rabbit were diluted using PBS and incubated at 37°C for 45 min, and 4′,6-diamidino-2-phenylindol (DAPI) was used for the nuclei staining for 5 min at RT. Before all operations, the samples were washed thrice with PBS. The cells were observed under a fluorescence microscope (Leica, Wetzlar, Germany).

### 2.8 50% tissue culture infective dose

Vero cells were inoculated into 96-well plates and cultured until a full monolayer formed. The viral solution was serially diluted 10-fold and inoculated into cells, with each concentration inoculated into eight wells. Cultures were maintained using DMEM containing 7 μg/mL trypsin. The cytopathic effect (CPE) was observed under a microscope 3 days after infection. The 50% tissue culture infective dose (TCID_50_) was calculated using the Reed–Muench formula (Yu et al., 2018).

### 2.9 Western blotting

Proteins were separated using 10% SDS-PAGE (Vazyme, Shanghai, China) and transferred onto a polyvinylidene fluoride membrane. The membranes were incubated for 1 h on a shaker at RT using 5% skim milk powder to prevent nonspecific binding. The membrane was incubated with HA- and GAPDH-specific primary antibodies for 2 h at RT or overnight at 4°C, and finally incubated with the corresponding source IRDye 800CW secondary antibody for 1 h at RT. Following containment, the membrane was washed thrice with TBST before each step. Results were observed using a Sapphire RGBNIR Biomolecular Imager (Azure Biosystems, United States).

### 2.10 Interaction prediction models

Tertiary structure prediction of the PEDV envelope protein (GenBank: WMT38786.1) was performed using Alphafold2. The E protein model with the highest accuracy was selected according to the predicted local distance difference test (pLDDT). The structure of PEDV envelope protein (GeneBank: WMT38786.1) was predicted using HADDOCK 2.4, which detected three subunits, namely, isocitrate dehydrogenase [NAD] β-subunit (IDHβ), mRNA-associated protein MRNP 41 (MRNP 41), and DNA-dependent RNA polymerase II subunit 9 (RBP9). These three host proteins were used for interaction prediction. The host proteins were obtained from the Protein Data Bank database. Optimal interaction models were screened and judged based on docking parameters, including the affinity indices of protein-ligand complexes, contact residue ratios, van der Waals forces, as well as electrostatic, binding, and desolvation energies. Proteins, Interfaces, Structures, and Assemblies (PDBePISA) was used to predict the polar bond type, accessible surface area, buried surface area, and folding free energy of the potential amino acid interaction sites in the interaction model. The three-dimensional structure of the interaction model was demonstrated using PyMOL, in which the polar bonds within 5 Å of the viral protein and the host protein were selected for amino acid interactions, and then the interaction sites with the highest confidence were derived from the PDBePISA results.

### 2.11 Statistical analysis

Data were analyzed using GraphPad Prism 7.0 and all data are expressed as mean ± standard deviation. A *t*-test was used to determine the statistical significance of the difference between the means. *p* < 0.05 was considered statistically significant.

## 3 Results

### 3.1 LC-MS/MS analysis of host proteins interacting with the PEDV E protein

**Figure 1:**
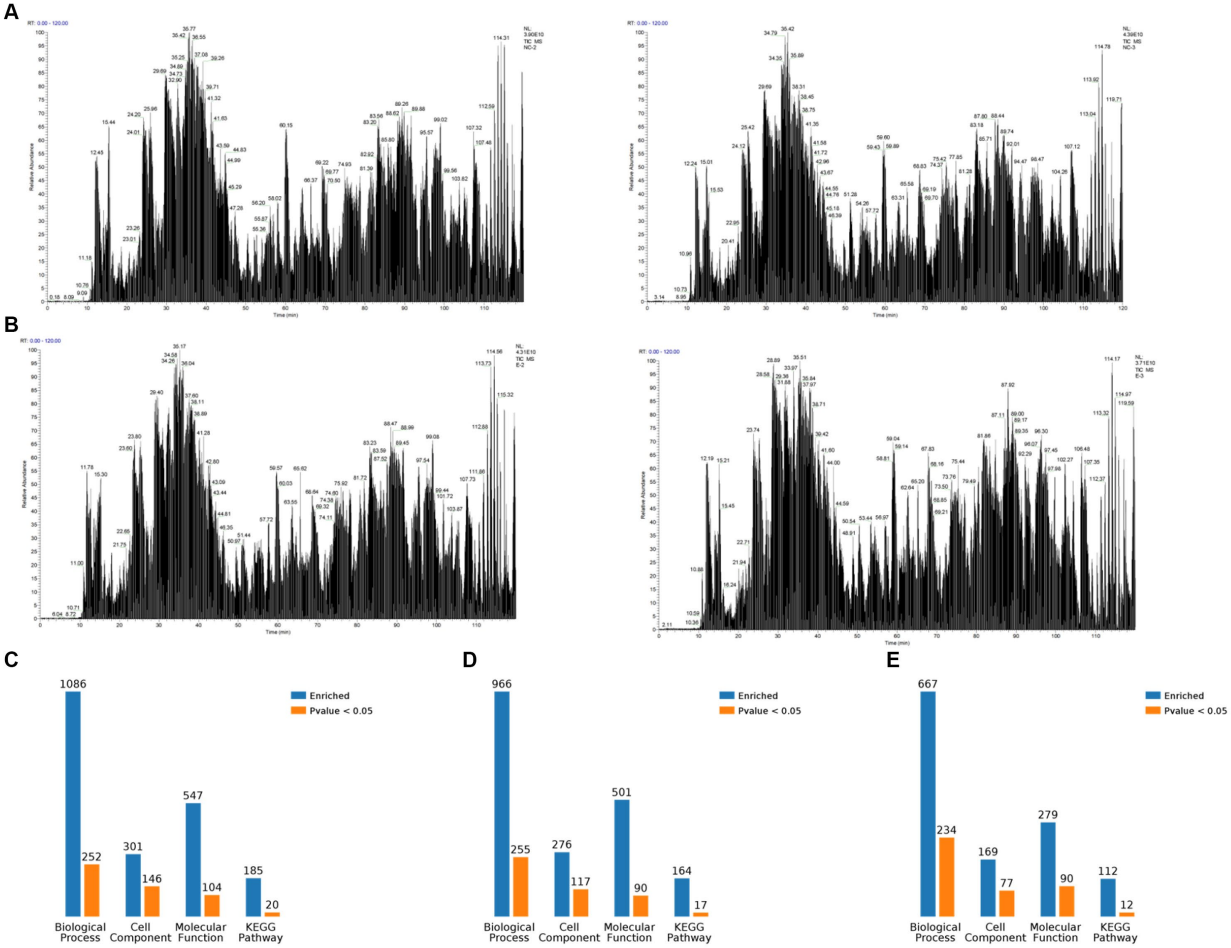
Overview of mass spectrometry data. **(A)** Total ion flow chromatogram obtained by Co-IP and mass spectrometry analysis after transfection of Vero cells witha plasmid expressing porcine epidemic diarrhea virus protein E, containing two replicates. **(B)** Total ion flow chromatogram obtained by Co-IP and mass spectrometry analysis after transfection of Vero cells with empty plasmid, containing two replicates. **(C)** Classification of E proteome-associated proteins. **(D)** Classification of relevant proteins in the empty control group. **(E)** Classification of relevant proteins in the E protein group compared with the empty control group.

The PEDV E protein expression and null carrier (NC) plasmids were transfected into Vero cells, and the PEDV E protein was evaluated using Co-IP. The processed samples were analyzed using LC-MS/MS, which generated raw MS results that were analyzed using MaxQuant software (1.6.2.10) to generate total ion flow chromatograms (Figures 1A,B). The total ion flow diagram shows high peaks with narrow widths, which indicated good separation efficiency of the liquid chromatography, and that the MS data were collected with good parallelism. A total of 835 differentially expressed proteins were enriched, of which 161 were significantly differentially expressed compared to those in the control. These differential proteins were enriched and analyzed through the KEGG pathway, resulting in 112 pathways, of which 12 were significantly different, including biosynthesis of amino acids, ribosome biogenesis in eukaryotes, RNA transport, RNA polymerase, dopaminergic synapse, carbon metabolism, Epstein–Barr virus infection, pyrimidine metabolism, spliceosome, adipocytokine signaling pathway, protein processing in endoplasmic reticulum, and influenza A. Simultaneously, differential proteins were subjected to Gene Ontology (GO) functional enrichment analysis based on biological process (BP), cellular component (CC), and molecular function (MF). The results showed significant enrichment in BP related RNA processing, RNA splicing, mRNA metabolic process, mRNA processing, and metabolic process. CC related intracellular part, intracellular, cell, cytoplasm, and cell part. MF related RNA binding, nucleoside phosphate binding, nucleotide binding, small molecule binding, ligase activity, forming aminoacyl-tRNA and related compounds (Figures 1C–E). The LC-MS/MS data was derived from previous research (Gao et al., 2023).

### 3.2 GO enrichment analysis of PEDV E interacting proteins

**Figure 2:**
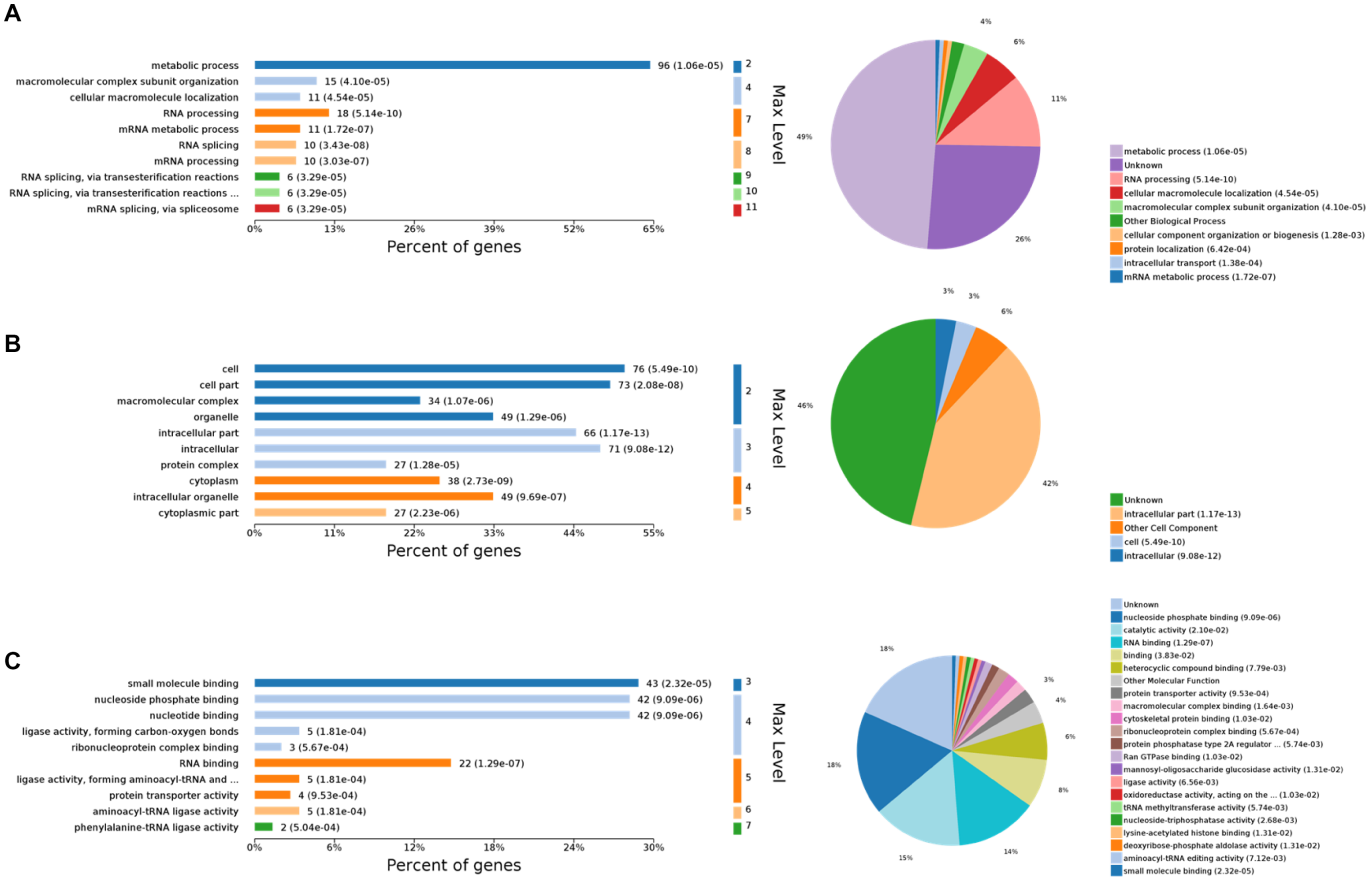
Gene Ontology (GO) functional analysis. The 10 most significant GO nodes are shown, and the biological processes that each protein is most likely to participate in are counted and represented in a pie chart. **(A)** Biological process. The horizontal axis represents the percentage of enriched proteins, and the number after each bar represents the number of proteins in that classification. **(B)** Cellular component. **(C)** Molecular function.

The 10 most significant GO nodes at different maximal levels were selected in the BP, CC, and MF categories, and the number and percentage of relevant proteins in each category were represented using bar charts. Based on the *p*-values, the most probable biological processes in which each protein was involved were determined, and pie charts were constructed based on the results (Figure 2). Compared to the control group, 667 BP functions were enriched, among which the metabolic process was the most important. It was also enriched with macromolecular complex subunit organization, cellular macromolecule localization, RNA processing, mRNA metabolic process, RNA splicing, mRNA processing, RNA splicing, via transesterification reactions, RNA splicing, via transesterification reactions with bulged adenosine as nucleophile, mRNA splicing, via spliceosome. The largest percentage of proteins were also involved in the metabolic process (49%), followed by RNA processing (11%), cellular macroscopic localization (6%), and macroscopic complex subunit organization (4%) (Figure 2A). CC was enriched to 169 related nodes, with significant differences in cell part, macromolecular complex, organelle, intracellular part, intracellular, protein complex, cytoplasm, intracellular organelle, and cytoplasmic part. Proteins related to the internal part are the most common (42%), followed by another cell component (6%), Cell (3%), and Internal (3%) (Figure 2B). MF was enriched to 279 nodes, with small molecule binding being the most important node. Significant differences are observed in nucleoside phosphate binding, nucleotide binding, ligase activity, forming carbon–oxygen bonds, ribonucleoprotein complex binding, RNA binding, ligase activity, forming aminoacyl-tRNA and related compounds, protein transporter activity, aminoacyl-tRNA ligase activity, and phenylalanine-tRNA ligase activity. The most relevant proteins for nucleoside phase binding were found to be 18%, followed by catalytic activity (15%), RNA binding (14%), binding (8%), heterocyclic compound binding (6%), other molecular function (4%), and protein transporter activity (3%) (Figure 2C).

### 3.3 KEGG pathway enrichment analysis of PEDV E interaction proteins

**Figure 3:**
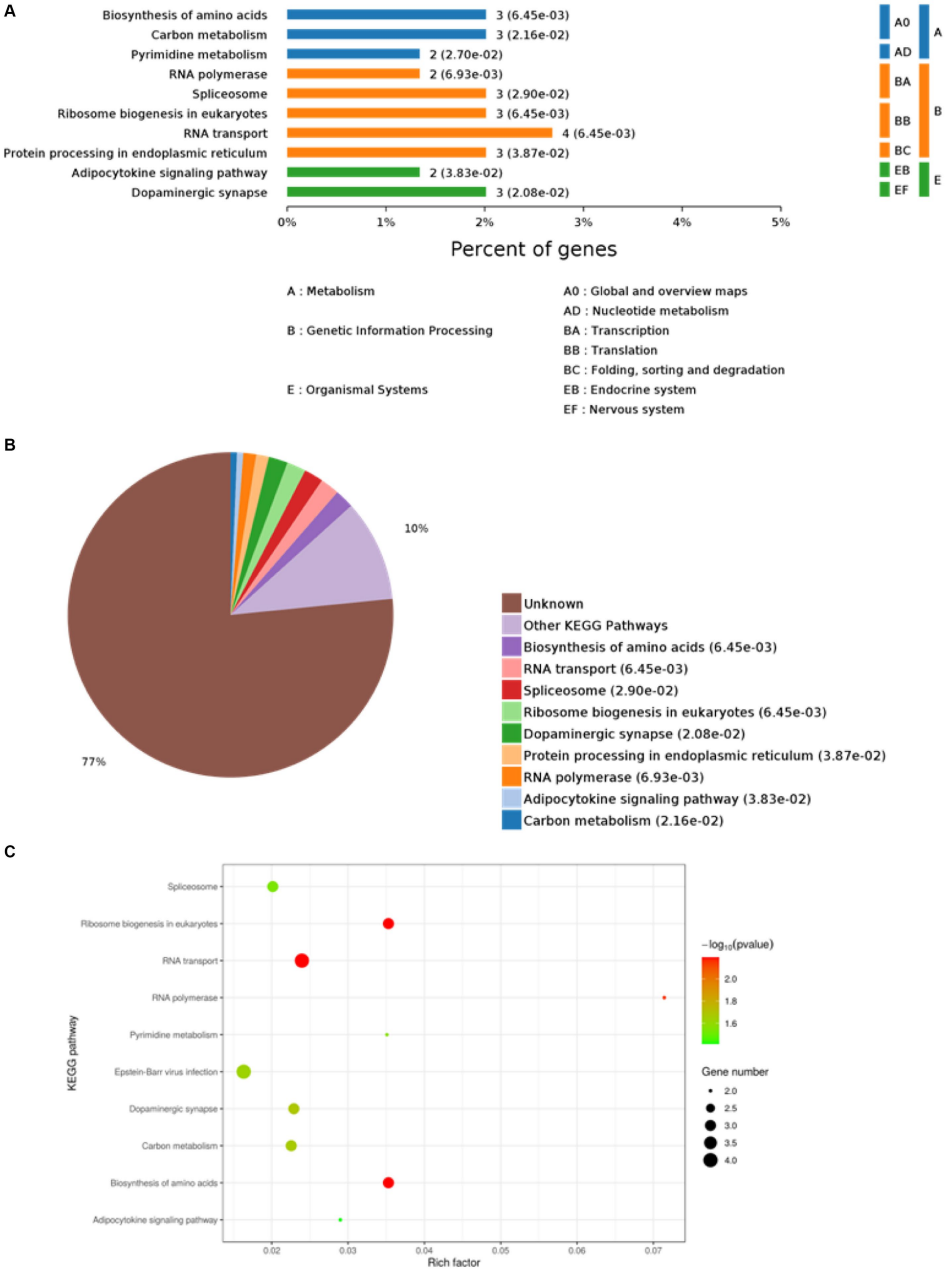
Kyoto Encyclopedia of Genes and Genomes (KEGG) analysis. **(A)** KEGG pathway enrichment category. **(B)** Classification and statistics of the KEGG pathway of interacting proteins. **(C)** Bubble diagram of the KEGG pathway for differentially expressed proteins.

The top 10 KEGG pathway enrichment categories with the most significant differences are shown, including biosynthesis of amino acids, carbon metabolism, pyrimidine metabolism, RNA polymerase, spliceosome, ribosome biogenesis in eukaryotes, RNA transport, protein processing in endoplasmic reticulum, adipocytokine signaling pathway, and dopaminergic synapse (Figure 3A). Based on the *p*-values, the biological processes in which each protein was most likely involved were identified, mainly including biosynthesis of amino acids, RNA transport, spliceosome, ribosome biogenesis in eukaryotes, dopaminergic synapse, protein processing in endoplasmic reticulum, RNA polymerase, adipocytokine signaling pathway, and carbon metabolism (Figure 3B). Host proteins that interacted with the E protein were mainly involved in amino acid synthesis, RNA transport, and spliceosomes. Finally, based on the bubble plot, we identified three interaction pathways: ribosome biogenesis in eukaryotes, RNA transport, and biosynthesis of amino acids (Figure 3C), considering the *p*-values, enrichment levels, and number of enriched proteins in each pathway.

### 3.4 Protein–protein interaction network analysis of PEDV E interacting proteins

**Figure 4:**
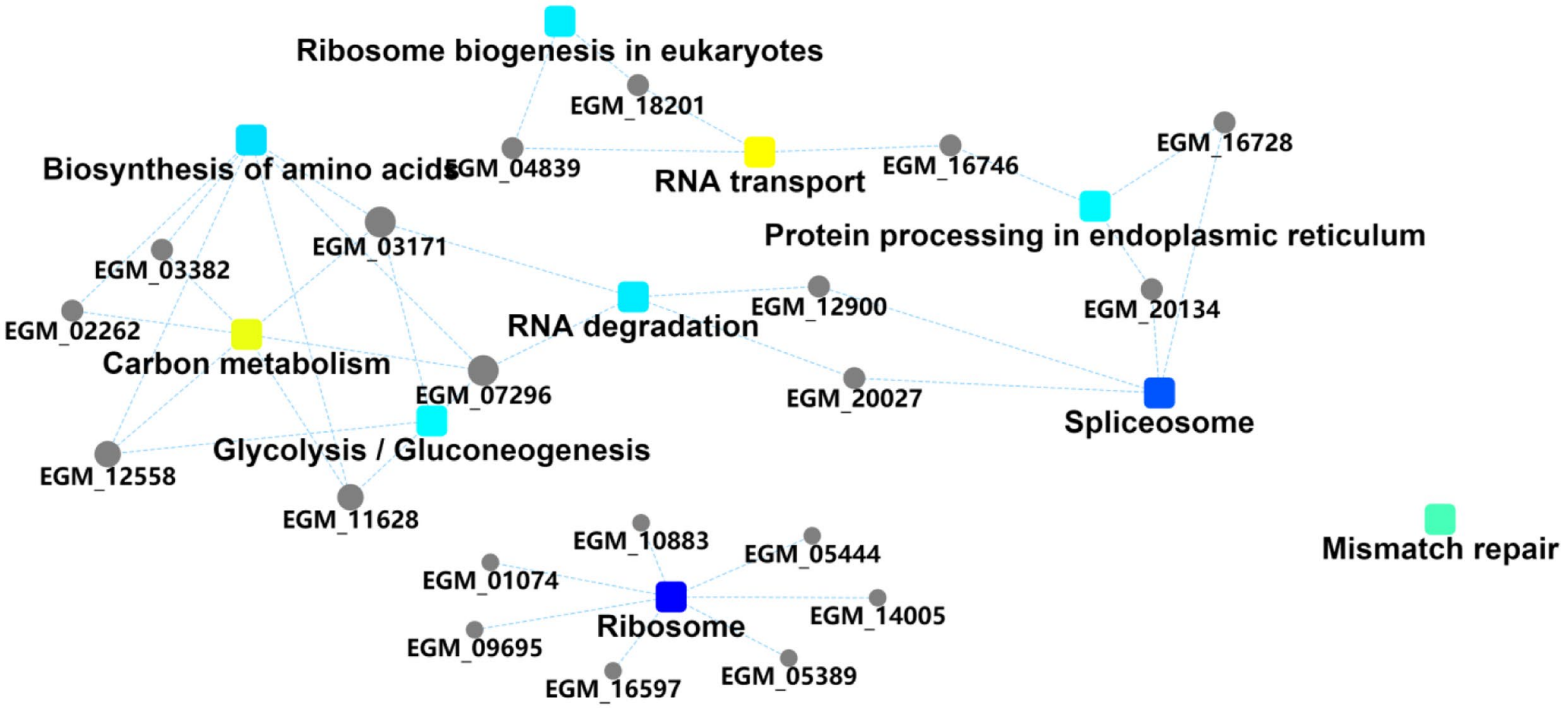
Protein interaction information. Squares represent GO/KEGG terms and circles represent genes/proteins.

The importance of the three pathways, namely eukaryotic ribosome biogenesis, RNA transport, and amino acid biosynthesis was again demonstrated by the differentially expressed protein interaction diagrams, which can be linked to three, four, and three host proteins, respectively, and can thus be linked to other biological processes (Figure 4). The ribosome biosynthesis in eukaryotes is mainly related to energy metabolism. RNA transport and biosynthesis of amino acids can also be interconnected through other proteins or pathways. We speculate that the PEDV E protein may regulate these pathways in the host to create favorable conditions for viral replication and proliferation.

### 3.5 Interaction validation between PEDV E and the three host proteins

**Figure 5:**
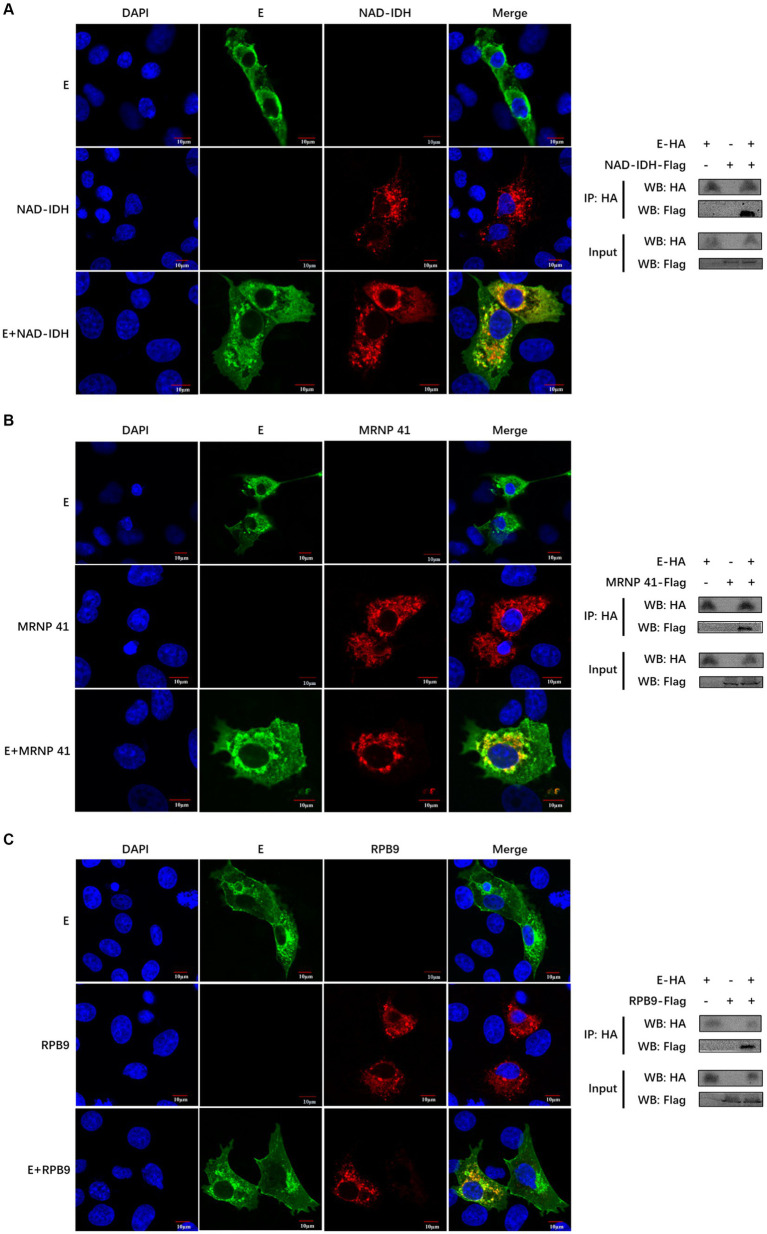
The PEDV E protein interacts with NAD-IDH-β, MRNP 41, and RPB9 host proteins. **(A–C)** The subcellular localization of PEDV E protein and host protein NAD-IDH-β, MRNP 41, and RPB9 in IPI-FX cells was detected by Confocal. The interaction between PEDV E protein and host protein NAD-IDH-β, MRNP 41, and RPB9 was detected by Co-IP.

We further validated the relationship between the PEDV E protein and the identified three pathways. We selected the only known proteins (NAD-IDH-β, MRNP 41, and RPB9) in each of the three pathways and verified their interaction with PEDV E proteins. The co-localization of PEDV E protein with host proteins NAD-IDH-β, MRNP 41, and RPB9 proteins, was detected using confocal analysis (Figures 5A–C). The PEDV E interaction with host proteins NAD-IDH-β, MRNP 41, and RPB9 was further demonstrated using the Co-IP results (Figures 5A–C).

### 3.6 NAD-IDH-β overexpression inhibits PEDV replication

**Figure 6:**
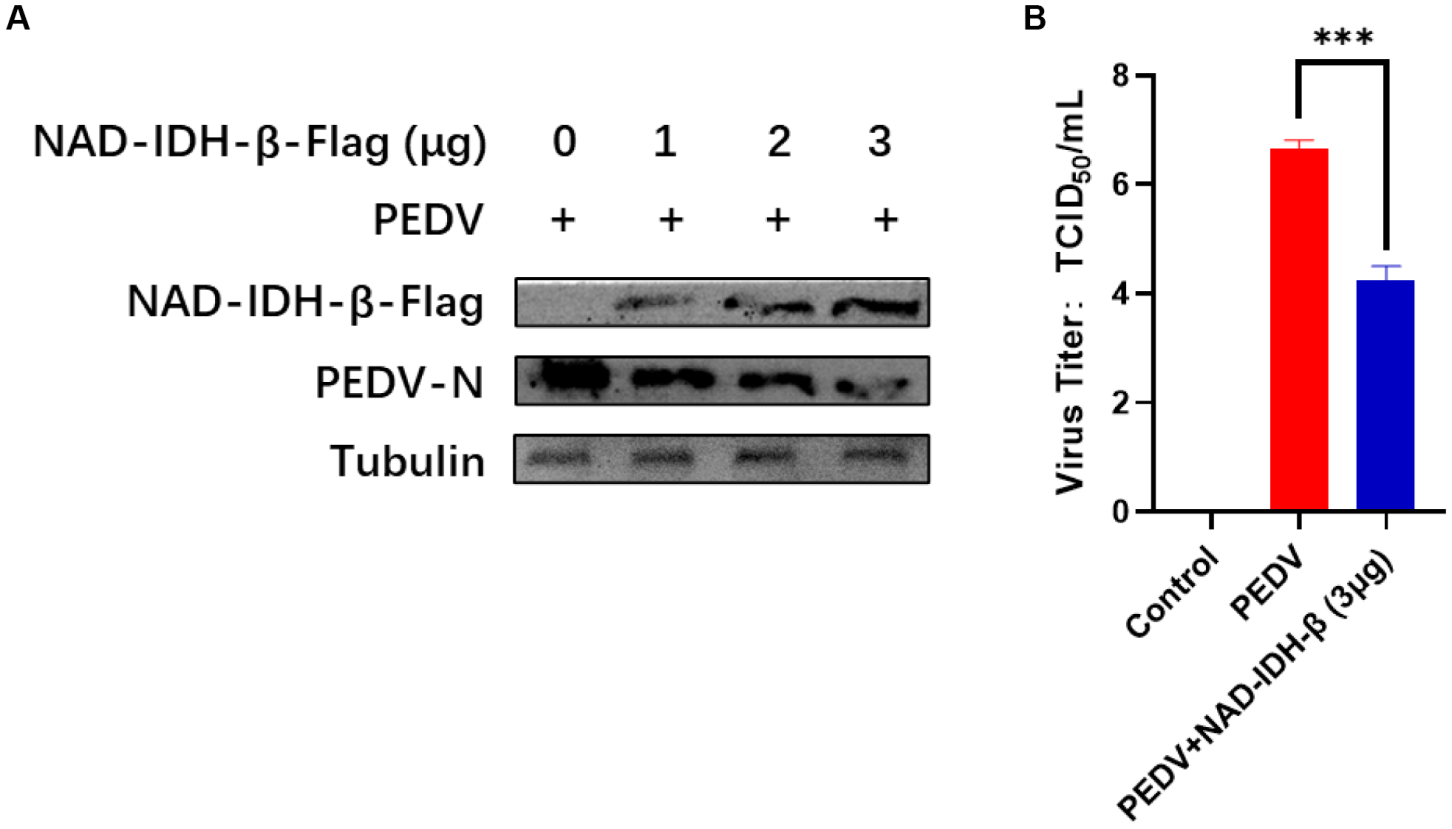
NAD-IDH-β overexpression inhibits PEDV replication. **(A)** After overexpression of 1 μg, 2 μg, and 3 μg NAD-IDH-β in IPI-FX cells, PEDV was infected and the expression level of PEDV N protein was detected. **(B)** After 3 μg NAD-IDH-β was overexpressed in IPI-FX cells, PEDV was infected and PEDV titer was detected.

To clarify the effect of the interacting protein NAD-IDH-β on PEDV replication, PEDV was inoculated in NAD-IDH-β plasmid-overexpressing IPI-FX cells, and the replication level of the virus was detected using TCID_50_ and western blot. The results showed that NAD-IDH-β overexpression significantly decreased the level of PEDV N protein expression (Figure 6A) and reduced the titer of PEDV (Figure 6B) compared to that of infection with PEDV alone, suggesting that NAD-IDH-β inhibits PEDV replication.

### 3.7 Interaction site prediction between PEDV E and the three host proteins

**Figure 7:**
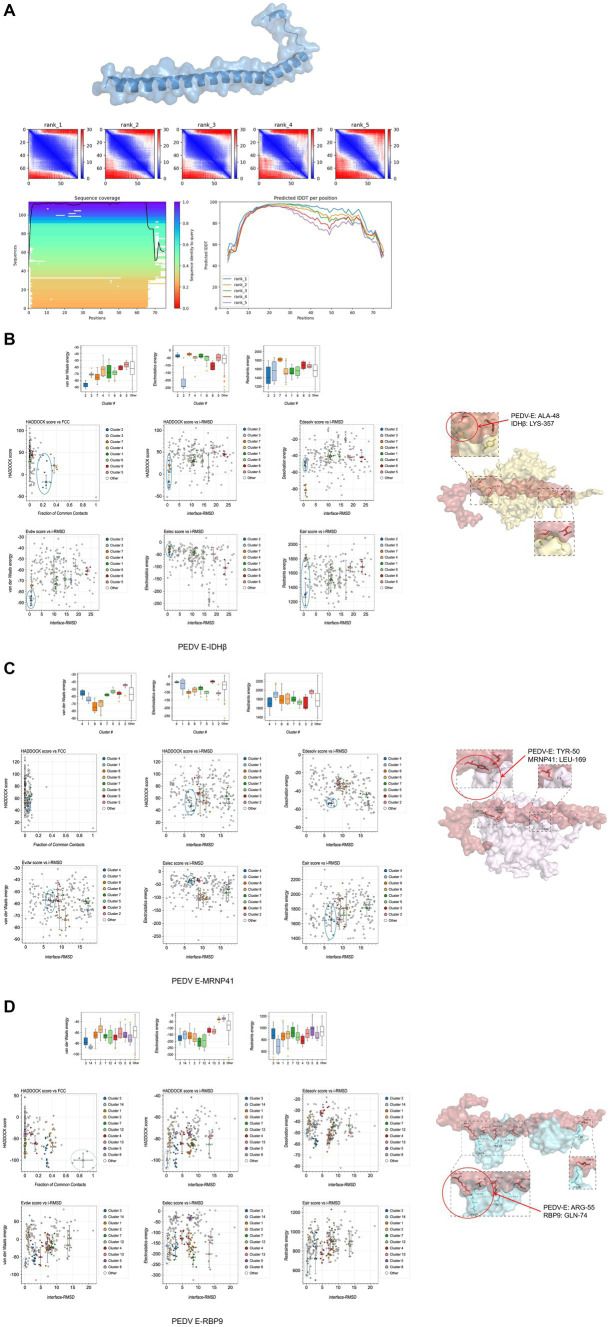
Predicted interaction sites of porcine epidemic diarrhea virus protein E (PEDV E) protein with host proteins NAD-IDH-β, MRNP 41, and RPB9. **(A)** E protein tertiary structure and optimal model prediction selection. (**B–D**) Protein interactions site prediction.

The interaction sites of PEDV-E with the host proteins NAD-IDH-β, MRNP 41, and RPB9 are unknown. The E protein tertiary structure was predicted and the optimal model was chosen (Figure 7A). Modeled interactions were predicted using HADDOCK, and a cluster of viral proteins classified by parameters such as conformation, interaction force, and potential energy during the docking of viral and host proteins. The results showed that the optimal prediction models of PEDV E protein with host NAD-IDH-β, MRNP 41, and RPB9 proteins were PEDV_E-IDHβ: cluster2, PEDV_E-MRNP 41: cluster4, and PEDV_E-RBP9: cluster14, respectively (Figures 7B–D).

Intercalation site selection was performed using PDBePISA and PyMOL, according to the optimal intercalation model in HADDOCK. In the table of PDBePISA, structure represents the amino acid residues and their corresponding positions, HSDC represents the polar bonds of amino acid residue interactions, accessible surface area (ASA) is the accessible surface area, buried surface area (BSA) is the buried surface area, and ΔG is the folding free energy. At the interaction interface, high ASA and BSA scores represented a large surface area exposed to the solvent and a large hidden surface area, in which the folding state of the protein was relatively stable and the free energy of folding was negative, indicating flexible and dynamic structure in the corresponding region. The predicted sites of amino acid interactions of PEDV E protein with host NAD-IDH-β, MRNP 41 and RPB9 proteins were PEDV_E-IDHβ: ALA-48 vs. LYS-357, PEDV_E-MRNP 41: TYR-50 vs. LEU-169, and PEDV_E-RBP9: ARG-55 vs. GLN-74 (Figures 7B–D). These findings provide a basis for studying the interactions between viruses and host proteins.

## 4 Discussion

The first PED outbreak occurred in 1972, and the causative agent was identified as PEDV in 1978 (Pensaert and de Bouck, 1978). Initially, there were no large-scale outbreaks of the disease, and it did not attract much attention (Su et al., 2020). However, in 2010, a highly pathogenic mutant strain with a piglet mortality rate of 100% emerged in China (He et al., 2022). Subsequently, countries such as the United States, Germany, and France successively reported highly pathogenic strains (Stevenson et al., 2013; Hanke et al., 2017). Consequently, PEDV has become a global epidemic and causes viral diarrhea in pigs, resulting in substantial economic losses. However, because of the rapid mutation and recombination of this strain, there is currently no effective vaccine or specific antiviral drug to treat this disease. The E protein is mainly involved in viral assembly and in viruses that lack the E protein, the titer is significantly decreased. However, whether the E protein is involved in other processes of the viral lifecycle or how host proteins can affect viral replication and proliferation remains unknown. In this study, we screened host proteins interacting with PEDV E protein using LC-MS/MS. PEDV mainly infects pig intestinal epithelial cells. However, the proliferation level of PEDV was low in pig intestinal epithelial cell lines, we chose Vero cells, which are highly sensitive and susceptible to PEDV infection (Hofmann and Wyler, 1988). The strain FS202201 we used was isolated from the Vero cell line (Sun et al., 2023). We verified that host NAD-IDH-β, MRNP 41, and RPB9 proteins interacted with PEDV E protein by confocal analysis and Co-IP, and found that host protein NAD-IDH-β overexpression inhibited PEDV replication.

In eukaryotes, the NAD-dependent IDHs (NAD-IDHs or IDH3) consist of three subunits (α, β, and γ), and exist as a (α2βγ)2 heterooctamer. Mammals have three IDH subtypes: mitochondrial NAD-IDH (IDH3), mitochondrial NADP-IDH (IDH2), and cytoplasmic NADP-IDH (IDH1) (Chen and Ding, 2023). The IDH1 mutation inhibits the virus-induced IFN antiviral response in glioma cells (Chen et al., 2023). IDH2 particles respond to oxidative injury and regulate lipogenesis and glycolysis (Filipp et al., 2012). Molecular or pharmacological inhibition of IDH2 greatly reduces the reductive TCA circulating flux (Zhang et al., 2021). In addition, IDH2 overexpression in lung cancer cells increased the reverse response of the TCA cycle; promoted anaerobic glycolysis, glutamine breakdown, and lactate production; and reduced cellular ATP content (Li et al., 2018). Notably, the Epstein–Barr virus (EBV) EBV-LMP1 protein enhances c-Myc binding to the IDH2 promoter and activates wild-type IDH2 via c-Myc transcription. The EBV-LMP1/c-Myc/IDH2WT signaling axis is critical for EBV-dependent metabolic changes and tumorigenesis (Shi et al., 2020). It has been reported that SARS-CoV-2 infection promotes the succinylation of several key enzymes in TCA, leading to the inhibition of cellular metabolic pathways (Liu et al., 2022). There is little research on the effect of PEDV proliferation on host energy metabolism. The mechanism by which E proteins interact with host proteins to regulate host metabolism and cause high pathogenicity deserves further investigation. In this study, NAD-IDH-β overexpression inhibited PEDV proliferation, suggesting that the E protein may regulate host metabolism by interacting with NAD-IDH-β, reducing host-provided energy for viral replication.

RPB9, a nonessential subunit of RNA polymerase II, mainly involved in DNA transcription and translation and controls transcription fidelity (Walmacq et al., 2009). RPB9 responds to UV-induced DNA damage, promotes the ubiquitination and degradation of stagnant RNAPII, and participates in transcriptional coupling repair via transcriptional elongation regulation (Sein et al., 2018). In the absence of RPB9, the mutation rate of the viral-like transcript is higher than that of the cellular transcript (Dissanayaka Mudiyanselage et al., 2022). Whether the interaction between RPB9 and E protein is involved in the transcription and translation process of PEDV virus, thereby improving its fidelity, deserves further investigation. Here, we found that the PEDV E protein interacted with the host protein RPB9, suggesting that RPB9 may promote viral proliferation by ensuring viral transcription fidelity. However, this hypothesis requires further investigation.

MRNP 41, an mRNA-binding protein, plays a role in cytoplasmic transport and directly or indirectly connects cytoplasmic MRNP to the cytoskeleton (Kraemer and Blobel, 1997). Furthermore, MRNP 41 and Rae1 are similar. In cancer, RAE1, which is driven by super-enhancers, exhibits high protein levels (Berkel and Cacan, 2023). Vesicular stomatitis virus inhibits the antiviral response of infected cells by inhibiting host gene expression. Its M protein-Rae1 complex serves as a platform to promote the interaction between the M protein and other factors involved in host transcription, which also support the transcriptional regulation of the Rae1-Nup98 complex (Rajani et al., 2012). MRNP plays a role in cytoplasmic transport, and whether its interaction with E protein interacts with other factors involved in host transcription or translation, thereby regulating the process of host transcription and translation system, deserves further investigation. Here, we found that the PEDV E protein interacts with host MRNP 41, suggesting that the PEDV E protein may regulate host transcription factor expression by interacting with host MRNP 41, thereby inhibiting host antiviral activity. However, this hypothesis requires further investigation.

In conclusion, we screened 161 host proteins that may interact with the PEDV E protein by combining Co-IP and LC/MS-MS. These proteins were enriched in three main biological processes: eukaryotic ribosome biogenesis, RNA transport, and amino acid biosynthesis. We verified the interaction of the PEDV E protein with a known protein contained in each of these three pathways, namely NAD-IDH-β, MRNP 41, and RPB9. NAD-IDH-β overexpression inhibited PEDV replication. In addition, the amino acid sites where the above host proteins interacted with PEDV-E proteins were predicted to be PEDV_E-IDHβ: ALA-8 and LYS-357, PEDV_E-MRNP 41: TYR-50 and LEU-169, and PEDV_E-RBP9: ARG-55 and GLN-74 by combining the conformation, force, and potential, respectively. This study provides a theoretical basis for the proliferation mechanism of PEDV and contributes to the development of vaccines and targeted drugs to treat PED.

## Data availability statement

The data presented in the study are deposited in the PRIDE repository, accession number PXD050352. The data is publicly available on the platform: www.ebi.ac.uk/pride/archive/projects/PXD050352.

## Author contributions

YQ: Writing – original draft, Writing – review & editing, Conceptualization, Methodology, Validation. YS: Data curation, Methodology, Writing – original draft. XZ: Formal analysis, Writing – review & editing. LG: Resources, Writing – review & editing. LY: Investigation, Project administration, Supervision, Writing – review & editing. BX: Software, Visualization, Writing – original draft, Funding acquisition.

## Footnotes

^1^ http://www.omicsbean.cn
